# A slanted-nanoaperture metal lens: subdiffraction-limited focusing of light in the intermediate field region

**DOI:** 10.1186/s40580-018-0165-y

**Published:** 2018-11-26

**Authors:** Yun Suk Jung, Myungji Kim, Yu Shi, Yonggang Xi, Hong Koo Kim

**Affiliations:** 1 Department of Electrical and Computer Engineering and Petersen Institute of NanoScience and Engineering, University of Pittsburgh, Pittsburgh, PA 15261 USA; 2 Present Address: ALKOR Semi, Cohoes, NY 12047 USA; 3 Present Address: Samsung Display, Yongin, South Korea; 4 Present Address: Google, Venice, CA 90291 USA

**Keywords:** Metal nanolens, Subdiffraction-limited focusing, Abbe limit, Super-lens, Surface plasmons, Vertical dipole, Radiation pattern, Nanoslit transmission

## Abstract

Diffraction of light limits the resolution of beam focusing with conventional lenses, as dictated by the Abbe limit, that is, approximately half the wavelength. Numerous techniques have been explored to overcome this limit. One of the most intensively explored approaches is to design a lens that operates in the near-field region, that is, with a focal length on the order of 10 nm, where evanescent fields can carry and project large in-plane wave-vectors (greater than free-space wave-vectors) to a focal plane. From a practical perspective, however, the requirement of such an ultra-short focal length puts too much constraint, since much longer focal length is commonly desired for intermediate or far-field operation. Here we report a method to beat the Abbe limit while operating with focal length greater than wavelength *λ*. Our approach is to tailor the radiation patterns of nanoaperture transmission by tilting aperture axes away from the surface of a metal film such that each slanted aperture transmits a highly directed, tilt-oriented beam onto a common focal point carrying maximal in-plane wave-vector components. The proposed nanoaperture array lens was fabricated by forming tilted nanoslits in a Ag, Al, or Cr film. We demonstrate minimal spot size of *λ*/3 (210-nm or 110-nm full-width half-maximum at λ = 633 nm or 325 nm, respectively) with 1–4*λ* focal length in air, beating the Abbe limit.

## Introduction

In beam shaping, a minimal spot size is determined by maximal spatial frequencies available on a focal plane. A variety of different approaches have been reported to overcome the diffraction limit in beam focusing [1–21]. They commonly aim to maximize the spatial frequencies on a focal plane, but with a variable degree of success and limitations. A major challenge outstanding in this field is to achieve both subdiffraction-limited resolution and far-field operation. First, in this Letter we briefly review the underlying principles of diffraction-limited focusing and the limitations entailed by presumed operating conditions, and explore a method to overcome the problem.

In conventional glass lenses a beam focusing function is performed via refractive transmission of light. In a microscopic view each atomic or molecular level scatterer diffracts incident light into spherical wavefronts, generating so-called Huygens wavelets. Since the spacing of these scatterers is much smaller than wavelength, the wavelets constructively interfere only to the forward direction (i.e., zero-order diffraction) and propagate to far-field without incurring any higher-order diffraction effect. As the wave propagates through lens medium, phase accumulation occurs with an amount proportional to lens thickness and refractive index. At the exit surface of a lens, wavelets emerge with a differing phase relationship, and they constructively interfere and propagate to a direction tilt-oriented from the surface normal.

One important point to note in this wavelet-based depiction of a refraction phenomenon is that the radiation pattern of each scattering element is intrinsically non-uniform in its angular distribution, that is, maximally transmitting to the forward normal direction and much reduced to tilt-oriented directions. This non-uniform angular distribution corresponds to an obliquity (inclination) factor of wavelet propagation [22]. Let’s consider a focal plane placed at far-field. The maximal frequency components available on the focal plane should come from the wavelets emanating from the most off-axis points of a lens, that is, the wavelets with the most glancing propagating (tilt-oriented) wave-vectors toward the focal point. Due to this obliquity factor, however, the high frequency components arrive at a focal plane with much lower intensity than those of normal transmission components. As a result, the maximum spatial frequencies available with a conventional lens are significantly smaller than the free-space wave-vector. The purpose of this paper is to show that the maximal frequency components on a far-field focal plane can be significantly increased by manipulating the radiation pattern of nanoaperture transmission in a metal nanolens.

## Transmission through a tilted nanoslit aperture

A metal nanoslit-array lens can be viewed as a discrete version of conventional glass lens [23]: an incident light transmits through each nanoslit and subsequently reradiates into its characteristic radiation pattern; the wavelets then constructively interfere to the zero-order diffraction direction, similar to the case of conventional dielectric interface described above. However, an important difference should be noted here: in metal lenses the radiation pattern of nanoaperture transmission can be easily altered for the benefit of enhancing the glancing-angle propagation components. Manipulating the nanoaperture transmission patterns corresponds to altering the obliquity factor of wavelet propagation in conventional glass lenses.

**Fig. 1:**
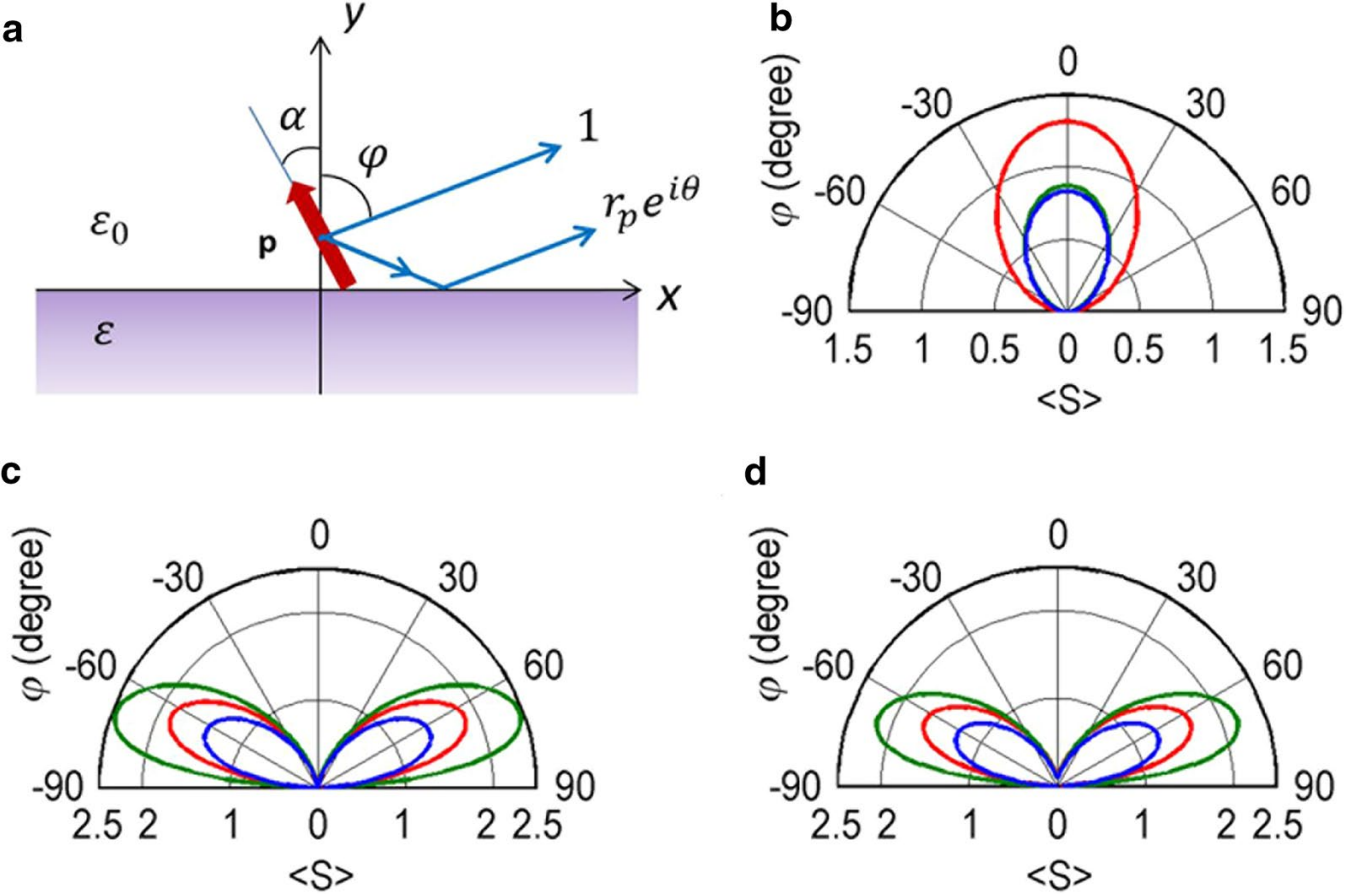
Dipole radiation patterns. **a** A tilted dipole placed on metal surface. The tilt angle α refers to the substrate normal. **b** Horizontal dipole (α = 90°). **c** Vertical dipole (α = 0°). **d** Titled dipole (α = 15°). Three different metals are assumed for substrate: Ag (red), Al (green) and Cr (blue). The radiation patterns calculated at 633 nm wavelength

In metal nanoaperture transmission, the opposing edges/corners of a nanoaperture opening on exit surface constitute a short dipole that reradiates an aperture-transmitted light into free space [23–25]. In conventional nanoapertures formed in a metal film, aperture opening is usually in-plane horizontal, and therefore the dipole axis is also aligned horizontal, being commonly referred to as a horizontal dipole. The far-field transmission pattern of this in-plane nanoaperture can be modeled as a horizontal dipole placed on metal surface. Similarly, a vertical nanoaperture can also be formed in a metal film by tilting the aperture opening to the normal direction and can be modeled as a vertically-oriented short dipole on metal surface [26–29]. As a generalization of this concept we assume a tilt-oriented short dipole on metal surface (Fig. 1a). The resulting radiation pattern at far-field can be described as a superposition of two co-propagating waves: a direct propagation from the short dipole and a reflected (therefore, phase-retarded) propagation from metal surface. The angular (*φ*) dependence of the radiation pattern, i.e., field intensity distribution, can be expressed as [29]:

1 $$\left\langle \varvec{S} \right\rangle \propto \left| {\left( {1 + r_{p} \left( \varphi \right)\exp \left( {i\theta \left( \varphi \right)} \right)} \right)\sin \varphi \cos \alpha + \left( {1 - r_{p} \left( \varphi \right)\exp \left( {i\theta \left( \varphi \right)} \right)} \right)\cos \varphi \sin \alpha } \right|^{2}$$

Here *α* is the tilt angle of dipole axis referring to the substrate normal, and *θ* denotes the amount of phase retardation of reflected transmission.

Figure 1b shows the radiation patterns of a horizontal dipole (*α* = 90°) calculated for three different cases of metal: Ag, Al and Cr. The following dielectric constants are assumed at 633 nm wavelength: *ε* = − 16.1 + *i*1.1 for Ag; − 56.5 + *i*21.2 for Al; − 1.2 + *i*20.8 for Cr [30–33]. The three cases show similar radiation patterns: maximally radiating to the normal direction and with angular width of ~ 80° full-width half-maximum (FWHM). Figure 1c shows a vertical dipole case (*α* = 0°). Note that the radiation patterns are highly directed: tilt-oriented to ~ 65° from the substrate normal and with significantly smaller FWHM (~ 43°) than the horizontal dipole case. Among the three metals, Al shows the largest amount of tilt. Figure 1d shows the case of a slanted dipole (*α* = 15°). The radiation patterns orient to the same directions as the vertical dipole case, although the intensity dropped a little bit. A slanted dipole is composed of two dipole components: horizontal and vertical dipoles. For relatively small tilt angles (*α* < ~ 15°) the radiation pattern is found to be mostly governed by the vertical dipole component.

**Fig. 2:**
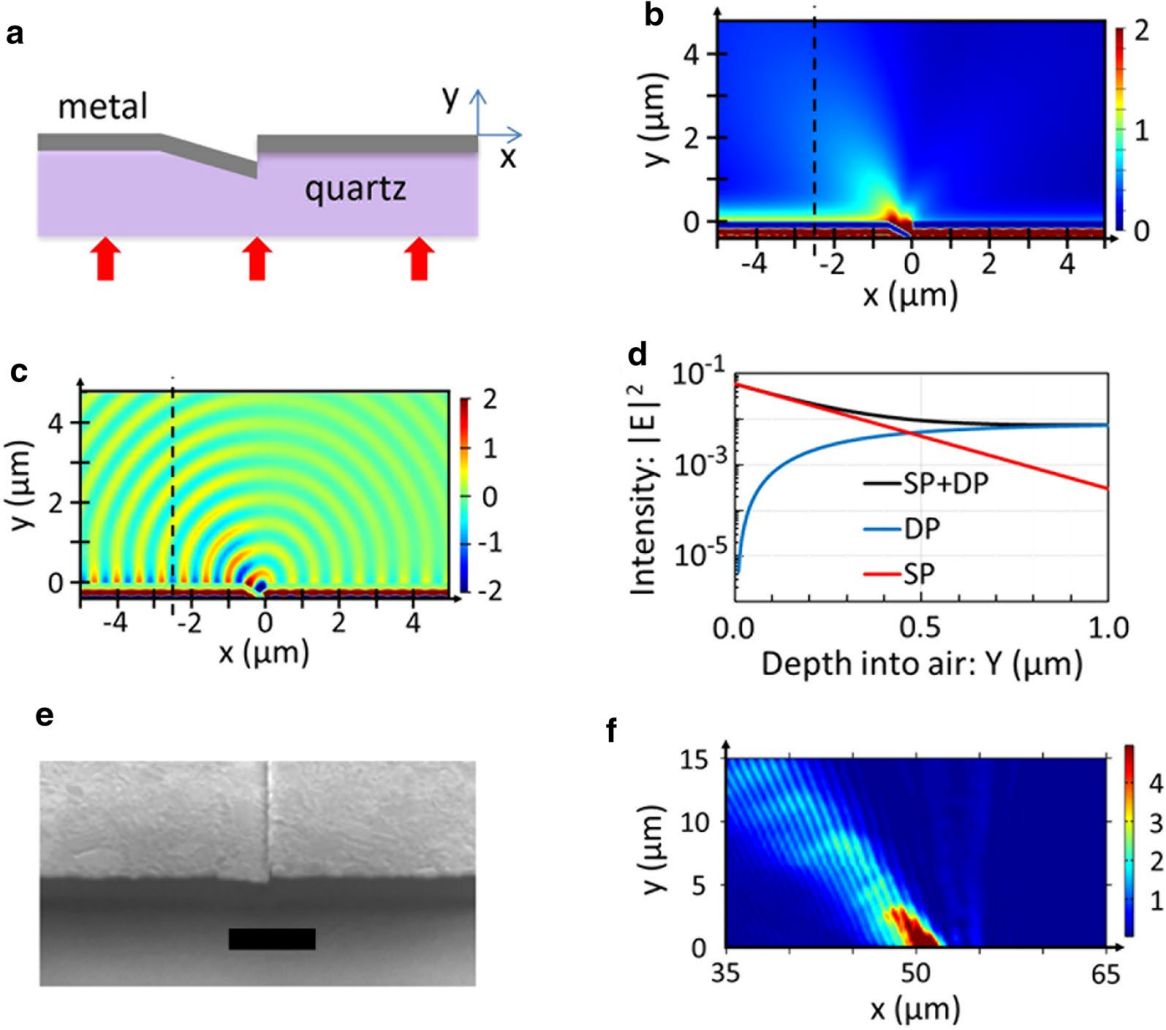
Transmission through a tilted nanoaperture. **a** Schematic cross-section of a tilted nanoslit formed in a metal film. A 633-nm TM-polarized light is incident from the bottom side. The transmission patterns calculated by FDTD simulation: **b** the distribution of magnetic field amplitude |*H*_*z*_|; **c** a snapshot image of magnetic field *H*_*z*_. Circular wavefronts emanate from the nanoslit aperture with tilted peak-intensity orientation. **d** The field intensity distribution scanned along the normal direction at *x* = − 2.5 µm (dashed). The total field (black) is decomposed into two parts: surface-plasmon field (SP, red) and free-space propagating component (DP, blue). **e** SEM image of a tilted nanoslit aperture formed in a Cr film (100- to 140-nm thick). Scale bar, 1 µm. **f** A beam profile measured by scanning an NSOM probe

Figure 2a shows a schematic cross-section of a slanted nanoslit simulated by finite-difference time-domain (FDTD) analysis: 60-nm slit width; 200 nm Ag thickness; 15° tilt angle of slanted step base. Spherical wavefronts emanate from the vertical nanoslit aperture and propagate to far-field with 47° tilt of peak-intensity orientation (Fig. 2b, c). This tilt angle is somewhat smaller (by 18°) than that of a model calculation of a vertical dipole on a flat horizontal surface (Fig. 1c). This discrepancy is ascribed to the fact that the step base itself in the FDTD simulation case is tilt-oriented (rotated) by 15°. The near-field region (*y* < ~ 200 nm; *x* < 0) reveals strong presence of surface plasmons (SPs) excited at slit edge.

**Fig. 3:**
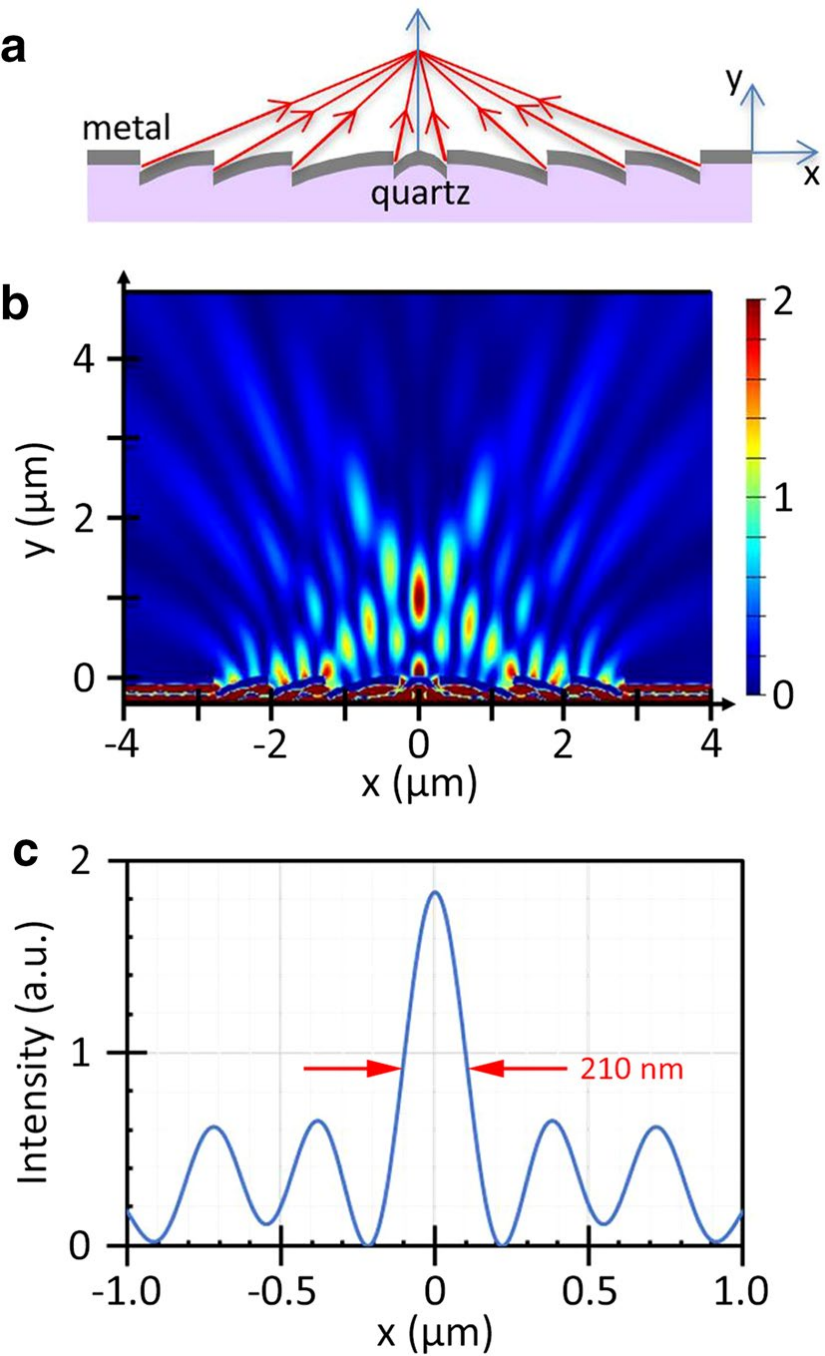
Beam focusing by a tilted nanoslit array lens. **a** Schematic cross-section of an 8-slit Ag nanolens designed for 2*λ* focal length at λ = 633 nm. **b** FDTD simulation of transmission pattern: field intensity distribution. **c** The intensity distribution scanned at *y* = 1.0 µm. The central peak shows 210 nm FWHM, corresponding to 0.33*λ*

The exit side of a nano-aperture generates two wave components: (1) surface plasmons (SP), i.e., a surface-bound wave propagating along the metal surface, and (2) free-space propagating waves emanating from a tilted nanoslit as a dipole (DP) radiation. It might be possible that the SP fields have reached the focal point with its large wave-vectors and contributed to the sub-diffraction-limited focusing observed in this work. In order to test this possibility we calculated the field distributions of each wave components (SP and DP) and compared their contributions to the focal point. Figure 2d shows a field intensity distribution scanned along the normal direction from metal surface to 1 μm in air at *x* = − 2.5 μm from a slanted nanoslit. This setting of slit location and observation point corresponds to the same distance between a focal point and the outer-most slit of an 8-slit lens described below (see Fig. 3). Two different field distributions are shown: one from the SP waves (SP, red) and the other one from the radiating components of aperture transmission (DP, blue). Here the total field distribution (SP + DP, black) is decomposed into two parts by taking the following steps: first, in the near-field region we assumed the dominance of SP component over the DP, and read the SP amplitude from the total field plot at metal surface (*y* = 0). The SP penetration depth was then read from a log-linear plot of the total field in the near-field region. The thus-extracted penetration depth is found to well match a theoretical calculation, that is, 390 nm for Ag/air interface. The DP field distribution is then calculated by subtracting the SP distribution from the total field.

Figure 2d reveals that the SP component is dominant only in the near-field region; a cross-over occurs at *y* = ~ 0.5 µm from the lens surface. At *y* = 1 µm, which corresponds to the focal point of an 8-slit lens (see Fig. 3b), the dipole radiation (DP) becomes 10 times stronger than the SP component (Fig. 2d). This confirms that the beam focusing at this focal point (*y* = 1 µm) is mostly contributed by the free-space propagating dipole radiation. (In Fig. 3b, the intense short fringes on metal surface are due to interference of counter-propagating SP waves. The evanescent nature of SP fields is evident from the observation that the fringe intensity sharply decays away from the surface with ~ 0.4 µm penetration depth and does not extend to the focal point at *y* = 1 µm.)

The tilt-oriented radiation pattern was experimentally demonstrated by employing a slanted nanoslit sample (Fig. 2e), which was fabricated in the following steps. First, a quartz substrate was focused ion-beam (FIB) etched to form a vertical step structure: 200-nm step height and 760-nm lateral span of a tilt-etched step base; this corresponds to 15° tilt angle of step base. A Ga ion beam was used with dwell time progressively increased for deeper etching along the tilt direction [26]. A 100- to 140-nm-thick Cr film was then deposited on the tilt-etched quartz substrate by thermal evaporation. The resulting slit width on the vertical sidewall is estimated to be 60–100 nm. Figure 2f shows the transmission pattern characterized by scanning an apertured near-field scanning optical microscope (NSOM) probe (Veeco Aurora 1720-00: 100-nm-thick Al coated; 80-nm aperture diameter). A TM-polarized light (633 nm wavelength, 1-mm beam diameter) was incident from the bottom side, and an NSOM probe was scanned on the exit side of the nanoslit aperture. The scan range was 60 µm on the horizontal direction and 40 µm in the vertical direction. The step size of scan was 50 nm and 157 nm in the horizontal and vertical directions, respectively. The NSOM-scan result shows 45° tilt of the main lobe (Fig. 2f) and compares well with the FDTD of Ag nanoslit (Fig. 2b, c).

## Beam focusing with slanted-nanoslit array lenses

Based on this single-slit result an array of slanted nanoslits is designed for a nanolens that can operate at intermediate/far-field, e.g., with 1–4*λ* focal lengths. Figure 3a shows a schematic cross-section of an 8-slit nanolens with focal length of 2*λ* at 633 nm wavelength in air ambient. The aperture location and orientation of each nanoslit is designed such that their radiation patterns orient to a common focal point and the wavefronts emanating from each slit arrive at the focal point in phase, i.e., constructively interfere there. The thus determined slit locations are: *x* = ± 0.26 µm; ± 1.26 µm; ± 2.04 µm; ± 2.78 µm. Here we also note that the location of the inner-most slit pair (at *x* = ± 0.26 µm) is determined taking into account the phase-retardation effect of SPs’ lateral propagation to the opposing slit, where they decouple into free space radiation.

Figure 3b shows FDTD simulation of transmission through the 8-nanoslit array Ag lens. The scan profile at the focal plane (*y* = 1.0 µm) is also shown. The central peak accounts for 30% of total beam power crossing the plane. The FWHM of this main peak is measured to be 210 nm. This corresponds to 0.33*λ*, smaller than the Abbe limit (~ λ/2). Each nanoslit pair generates two propagating waves with the following in-plane wave-vectors when projected to a focal plane: ± *k*_0_sin*θ*. The resulting interference pattern can be expressed as:

2 $$\left| {e^{{i(k_{0} \sin \theta )x}} + e^{{ - i(k_{0} \sin \theta )x}} } \right|^{2} \propto \cos^{2} \left( {\left( {k_{0} \sin \theta } \right)x} \right) .$$

For each slit pair the corresponding fringe width (π/2*k*_0_sin*θ*; FWHM) is calculated to be 167 nm, 175 nm, 199 nm, and 607 nm, counting from the outer-most slit to the inner ones. The resulting beam profile of the 8-slit nanolens is found to broaden a little bit (to 210 nm FWHM of the central peak: see Fig. 3c), but this width is still significantly smaller than the Abbe limit (~ 316 nm) at 633 nm wavelength.

**Fig. 4:**
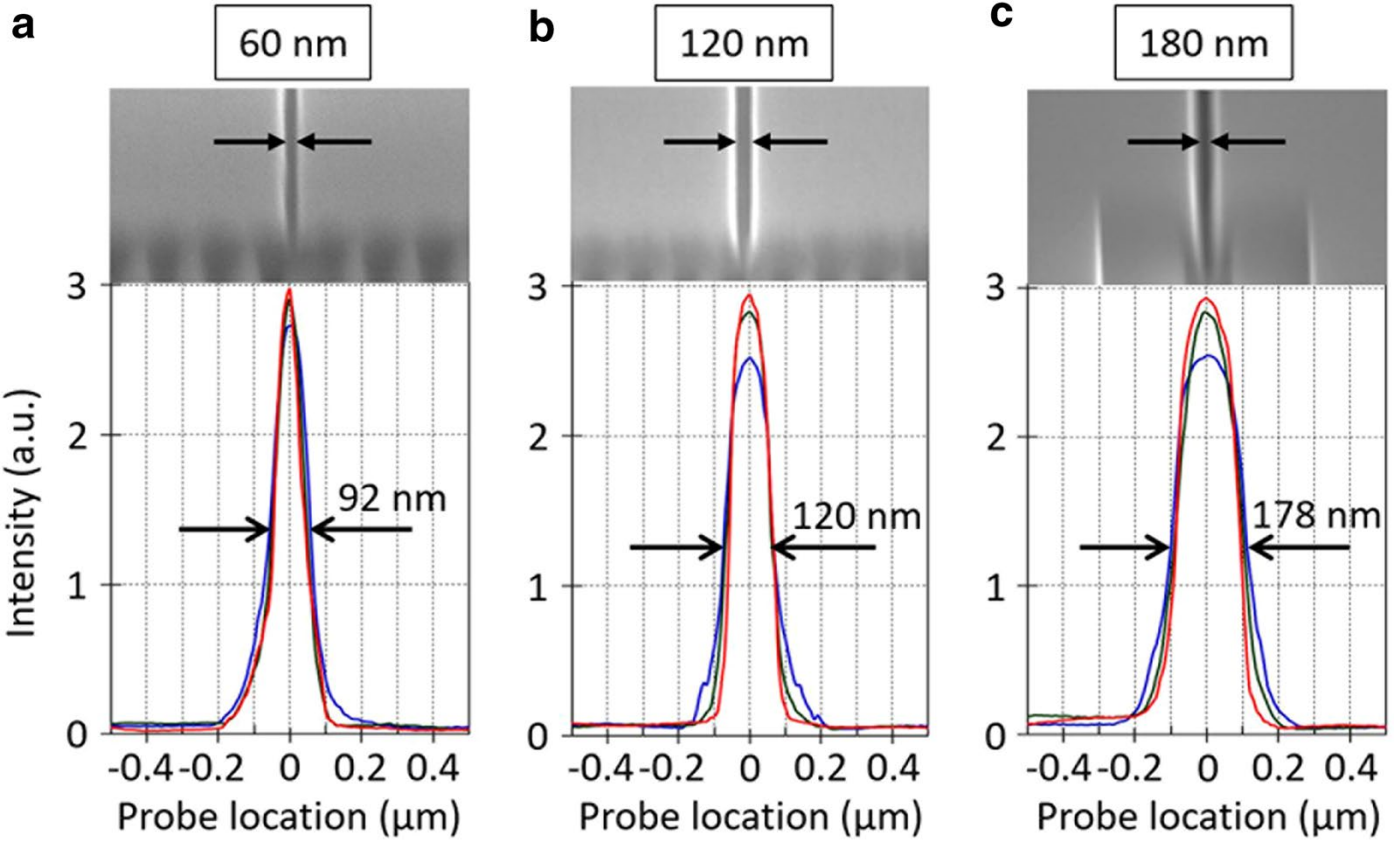
Spatial resolution of NSOM scanning. A single horizontal nanoslit (slit width of 60 nm, 120 nm, or 180 nm) was formed in a Cr film (65-nm thick): see top panels for SEM images. The NSOM scan profiles at *y* = 0.16 µm (red), 0.31 µm (green), and 0.47 µm (blue) at 633 nm wavelength: see bottom panels. **a** 60-nm slit sample showing 92 nm FWHM. **b** 120-nm slit sample showing 120 nm FWHM. **c** 180-nm slit sample showing 178 nm FWHM

Accurately measuring the minimal spot size at a given focal plane is crucially important in our efforts to draw a solid, unambiguous conclusion on whether we beat the Abbe limit or not. The accuracy of our NSOM measurement was tested with reference samples that were fabricated for this study. A single horizontal nanoslit structure was fabricated by FIB etching of a Cr film (65-nm thick) deposited on quartz substrate, with three different slit widths: 60 nm, 120 nm and 180 nm; see Fig. 4a–c, respectively. Figure 4 (bottom panel) shows NSOM-scan profiles measured at three different heights (*y* = 0.16 µm, 0.31 µm, and 0.47 µm) at 633 nm wavelength: the step size of horizontal scan was 10 nm. The three scan profiles show the same FWHM values for each slit: 92 nm for 60-nm slit, 120 nm for 120-nm slit, and 178 nm for 180-nm slit. Note the excellent agreement between NSOM and SEM measurements for the 120-nm and 180-nm slit samples and the large discrepancy (92 nm by NSOM versus 60 nm by SEM) for the 60-nm slit sample case. This measurement result suggests that the resolution of our NSOM scan is better (smaller) than 120 nm.

**Fig. 5:**
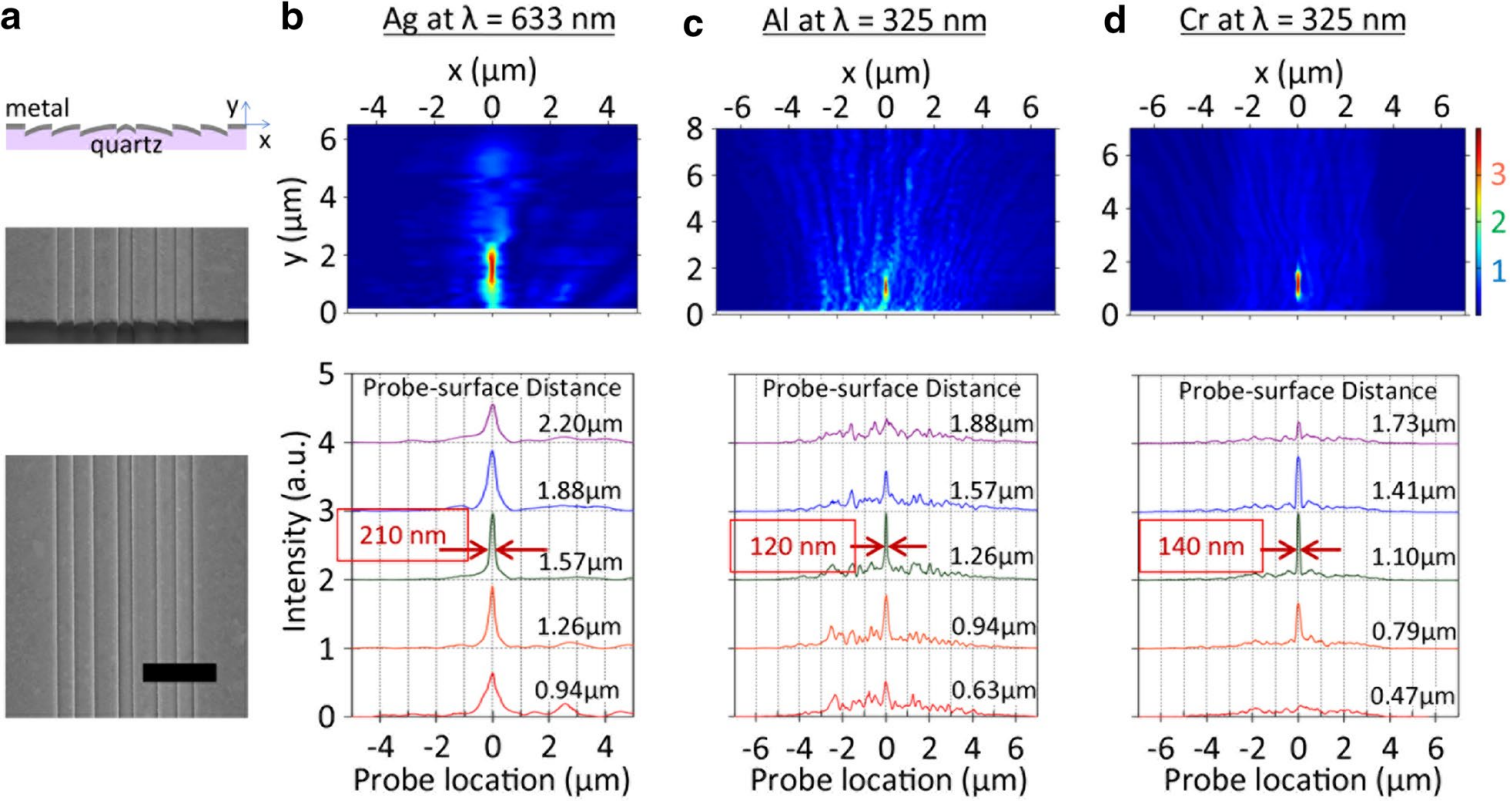
Subdiffraction-limited focusing of light with a tilted nanoslit array lens. **a** SEM image of an 8-slit Ag lens. Scale bar, 3 µm. **b** Ag lens characterized at 633 nm wavelength. NSOM scan image (top) and scan profiles (bottom). A minimal spot size is measured to be 210 nm FWHM at *y* = 1.57 µm. **c** Al lens at 325 nm wavelength. A minimal spot size of 120 nm is observed at *y* = 1.26 µm. **d** Cr lens at 325 nm wavelength. A minimal spot size of 140 nm is observed at *y* = 1.10 µm

The designed nanoslit array lens structure was fabricated on quartz substrate by FIB etching and followed by metal deposition: Fig. 5a for SEM images (top: side view; bottom: plan view). Note the symmetric configuration of aperture orientations across the lens axis. After FIB etching of four tilted-steps on one side, the same FIB process was repeated to complete the other four steps with an opposite tilt orientation. The overall dimensions of an 8-slit array lens are: 5.6-µm width (lateral span of 8-slit array) and 50-µm length (slit length). An Ag (150 nm), Al (150 nm) or Cr (80 nm) film was then deposited by thermal evaporation with an incident flux normal to the substrate. The resulting slit width on the vertical sidewall is estimated to be 60–100 nm.

The beam focusing function of the fabricated nanolenses was characterized by NSOM technique. A TM-polarized light (633 nm or 325 nm wavelength; 1 mm beam diameter) was incident from the bottom side, and an NSOM probe was scanned in the horizontal direction in the following window: − 10 µm < *x* < + 10 µm; 0 µm < *y* < 7 µm. The scan step size was 10 nm for *x*-direction and 157 nm for *y*-direction. For the high resolution (10 nm step) scan in the *x*-direction, a piezo servo-controller (PI E-665.CR) and a nano-stage (PI P-621.1CD) were used with 5-ms integration time, 150-µs dead time and 200-ms step delay. For the *y*-axis scan, a piezo driver and stage (Melles-Griot 17 PCS 001 and NanoBlock 3-axes Flexure Stage) were used with a 157-nm step size. An aperture-NSOM probe was initially brought to the proximity of a sample; the probe was further moved forward touching the sample surface. After this zero-point checking the probe was retracted back by one step (157-nm step size) and a scanning ensued. This NSOM scanning experiment was performed on a vibration isolation table.

Figure 5b shows the result with an 8-nanoslit Ag lens measured at 633 nm wavelength. Scan profiles are shown at several probe-surface distances (bottom panel). In the intermediate field region (*y*–*λ*) the beam profile becomes simpler and narrower. A minimal spot size (FWHM) is measured to be 210 nm at *y* = 1.57 µm. This corresponds to *λ*/3 FWHM, smaller than the Abbe limit.

Figure 5c, d shows the NSOM-scanned transmission patterns of 8-slit Al or Cr lenses designed for 4*λ* focal length at 325 nm wavelength. A focal plane is observed at 1.26 µm (for Al) or 1.10 µm distance (for Cr) from the lens surface, corresponding to 3.9*λ* or 3.4*λ* focal length, respectively. The minimal spot size (FWHM) is measured to be 120 nm (for Al) or 140 nm (for Cr). These FWHM values correspond to 0.37λ or 0.43λ, again smaller than the Abbe limit. It is interesting to note that the minimal spot size of the Al lens is slightly smaller than that of the Cr lens. This is ascribed to the fact that the radiation pattern of a vertical dipole on Al surface is more tilt-oriented (not shown in this paper) and therefore providing larger in-plane wave-vectors than the Cr case.

The dominance of the aperture radiation contribution over SP’s to sub-diffraction-limited focusing is confirmed in this experiment with a Cr lens characterized at 325 nm wavelength (Fig. 5d). The Cr/air interface poorly supports SPs at this wavelength: the propagation length and the penetration depth into air are calculated to be 0.5 µm and 190 nm, respectively, assuming ε = − 3.5 + *i*8.4 [31–33]. With such a small penetration depth and propagation length, a SP contribution to the focal point at *y* = 1 µm is expected to be negligible, and yet the Cr lens demonstrates sub-Abbe-limit focusing (140 nm FWHM < 163 nm of Abbe limit). This supports our conclusion on DP fields’ dominant contribution to the subdiffraction-limited focusing at far-field of our lens.

We also calculated the transmitted power of a horizontal nanoslit and a slanted nanoslit formed in a 150-nm-thick film with the same amount of aperture opening (slit width of 60 nm). At 633 nm wavelength, the total transmitted power of the slanted nanoslit is found to be ~ 45% of the horizontal nanoslit’s. This difference is partly ascribed to a smaller effective-aperture-area of a slanted nanoslit seen by an incident beam. The transmission efficiency of the horizontal nanoslit is estimated to be 120%. Here the transmission efficiency is defined as the ratio of total transmitted power to the incident power that falls on the aperture area, that is, the throughput power is normalized by the aperture area seen from the incident beam direction. It is interesting to note that the transmission efficiency is greater than 100%. This implies the nanoslit transmission involves a funneling effect occurring at aperture edges on the incident side [34]. In the case of the 8 slanted nanoslit array lens (Fig. 3a), the transmission efficiency is estimated to be 4.8%. In this estimation, the total transmitted power is normalized by the input power that falls on the entire slit array, that is, 5.6 µm wide area. Considering the relatively large spacing between nanoslits, the reduction of overall transmission is explained by the fact that the total, effective aperture areas of 8 slits account for a small portion of the total lens area.

## Comparison with super-oscillatory lenses

It is highly instructive to compare the results and mechanisms of this work with those of another interesting technique that also demonstrates subdiffraction-limited focusing at far-field. It is well known in literature that a band-limited function can oscillate faster than their fastest Fourier components, generating so-called super-oscillations [35, 36]. According to this theory, an arbitrarily small (i.e., narrow) focal spot can form at far-field of a properly-designed super-oscillatory lens, which looks to violate the Fourier transform relationship. A recent experimental work also demonstrates 0.45*λ* FWHM with 25*λ* focal length by employing a nanoaperture-array metal lens designed by this super-oscillation principle [37]. In fact, this seemingly paradoxical behavior does not contradict the Fourier relationship and can be explained as follows.

First, in Fourier theory the spatial frequency components refer to the entire beam profile defined in a global perspective, whereas in the super-oscillation theory they are locally defined in a narrow region. In this local frequency concept, presence of a narrow peak implies involvement of local large wave-vectors there, implying a rapid change of optical phase in that region. The local wave-vectors in this super-oscillatory region commonly exceed 10 times the free-space wave-vector [36]. This super-oscillation is an interference effect of band-limited propagating waves and requires a precise and delicate control of both amplitude and phase of Fourier components. It is important to note that a superposition of these propagating waves results in high-intensity side lobes that surround the narrow peak of much lower intensity in the central region. There also exists a tradeoff between the narrow peak and side lobes: the narrower the central peak the stronger the intensity of side lobes. As the central peak becomes narrower, the sidebands shift closer to the center, making it difficult to separate them. For example, the intensity of a central peak with *λ*/5 FWHM is 10^−7^ times weaker than that of sidebands, and the peak is separated from sidebands by 0.5λ distance [36].

By contrast, our approach is to manipulate the angular distribution of aperture radiation by tilting the aperture axis such that the transmitted power is directed into a focal spot at far-field with a reduced amount of solid angle. The directed beaming nature of the slanted nanoslit is the core difference with the conventional nanoslit structure case, in which a horizontally-open nanoslit radiates into the normal direction with larger solid-angle. In our nanoslit-array lens the aperture transmission from the outermost slit is the most glancing-angle propagating, and therefore it carries the largest wave-vector component to a focal plane. It should be noted that the aperture transmissions emerging from the slits are all in-phase. In general, this phase relationship can be included as a design parameter in a way similar to designing a super-oscillation lens [36]. In the current work, this phase factor was not taken into further optimization. Instead, by manipulating the obliquity factor only we achieved 0.33λ FWHM of focal spot and 1–4λ focal length. This focal spot accounts for 30% of total transmitted power, and no major side lobes appear on the focal plane. To the authors’ knowledge, this spot width is even smaller than the best result (0.45λ FWHM) of beam focusing with a super-oscillation lens reported in the literature [37].

Note that the focal length of our lens scales up with the lateral dimension of a lens, that is, the size of nanoslit array, and the maximum glancing angle of aperture transmission. In the present work we obtained 4λ focal length from a 5.6-µm size array lens. By employing a larger size lens the focal length can be further increased, for example, 40λ focal length from a 56-µm lens while maintaining the same level of sub-Abbe-limit focusing. This scalability can be compared with the super-oscillatory lens that demonstrates 25λ focal length from a 40-µm diameter lens [37]. It should also be noted that the focal spot size can be proportionally scaled down to much smaller width by employing a high-index immersion optics configuration. Ref [38] reports an experimental work that demonstrates sub-50 nm FWHM focal spot of a super-oscillatory lens placed in high-index solid-state immersion ambient (GaP with a refractive index of 3.72).

Table 1 summarizes comparisons of the three different nanolenses that demonstrate sub-Abbe-limited beam focusing.

**Table 1:** Comparison of a slanted nanoslit array lens with superoscillatory lenses: sub-Abbe-limit beam focusing at far-field

Type of lens and ambient	Materials	Size (µm)	Working wavelength, λ (nm)	Spot size, FWHM	Focal length	Power ratio	References
Slanted nanoslit array in air	Air/Ag/glass	5.6	633 325	210 nm (0.33λ) 120 nm (0.37λ)	1.6 µm (2.5λ) 1.3 µm (4λ)	4.8%	This work
Super-oscillatory in air	Air/Au/glass	40	405	185 nm (0.45λ)	10 µm (25λ)	–	[37]
Super-oscillatory in GaP (n = 3.72)	GaP/metal/glass	42	473	57 nm (0.12λ)	5 µm (11λ)	1.8%	[38]

## Beam focusing effects of an all-dielectric slanted-nanoslit array lens

**Fig. 6:**
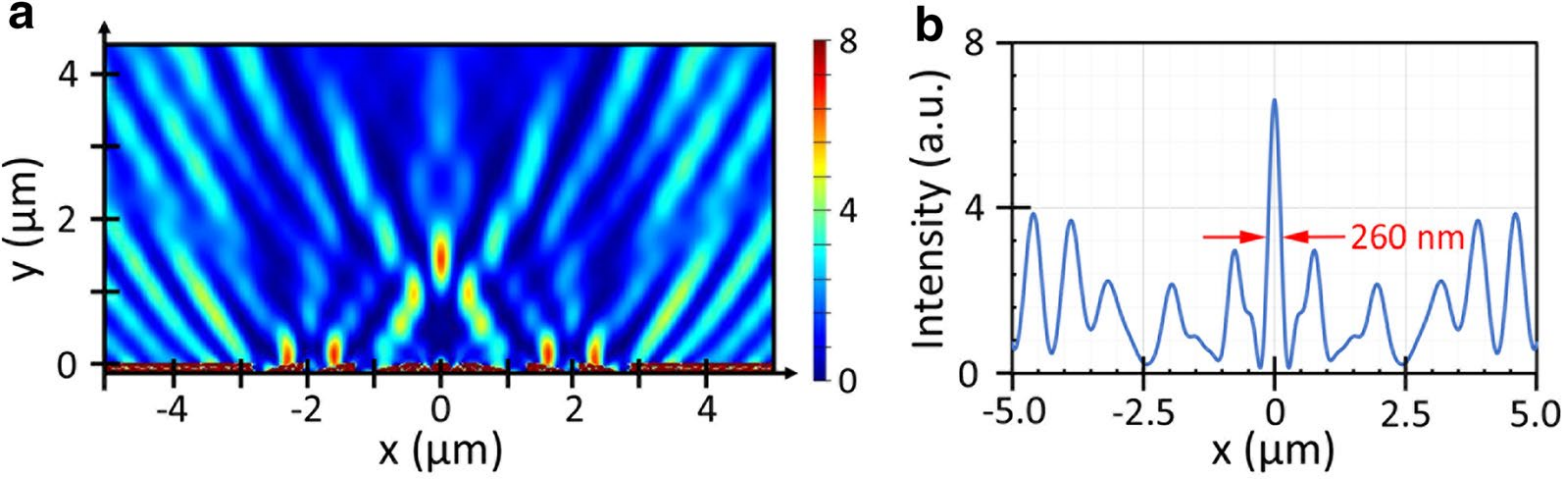
Beam focusing effects of an all-dielectric (Si/silica) slanted-nanoslit-array nanolens. **a** FDTD simulation of transmission pattern simulated at 633 nm wavelength. The same structure and dimensions are assumed as the Ag lens case (Fig. 3). **b** The intensity profile scanned at the focal plane

As an alternative to metallic metamaterials, all-dielectric metamaterials hold promising potential for nano-optic applications, where energy loss is an important factor that would limit device performance [39]. In the present work we also simulated beam-focusing effects of an all-dielectric slanted-slit array nanolens that employs Si instead of Ag for nano-aperture formation. Figure 6 shows a FDTD analysis of beam focusing effect of a dielectric nanolens at 633 nm wavelength. Here we assumed the same structure and dimensions as the 8-slit Ag lens discussed above (Fig. 5a): a Ag layer (150 nm thick) was simply replaced by a Si layer of the same thickness. Figure 6a shows an overall transmission pattern, and Fig. 6b an intensity profile scanned at the focal plane (*y* = 1.5 µm). Interesting observations can be made as follows. First, the FWHM of the center peak is measured to be 260 nm, corresponding to 0.41λ. This width is somewhat larger than the Ag case (210 nm), but still smaller than the Abbe limit. The entire region is crowded with many interference fringes of high intensity. In the scan profile, these fringes appear as intense side-lobes and they account for a major portion of the total transmitted power.

The many-fringe nature of this Si lens’s transmission pattern is explained as follows. First, two types of waves co-exist in the transmission side: direct transmission through a Si layer and aperture transmission through 8 tilted-nanoslits formed in Si. Two different types of interference patterns will then be generated: (1) between direct transmission and aperture transmission and (2) between two aperture transmissions coming from opposing directions. Type 1 interference involves planar wavefronts (of direct transmission) and circular wavefronts (of aperture transmission). The resulting fringe pattern evolves into a parabolic profile at far-field [40]. Type 2 interference involves two circular wavefronts arriving from opposing directions, and produces the central peak and satellite peaks at the focal plane. The direct transmission through a Si(150 nm)/SiO_2_ layer is estimated to be 63% (transmittance). It is interesting to note that the central peak is of similar intensity with side lobes. This implies that the transmission through a Si nanoslit aperture is reasonably strong. In brief, the following conclusions can be drawn from this simulation study: a far-field sub-diffraction-limited beam focusing is demonstrated with an all-dielectric (Si/SiO_2_) nanolens that employs a slanted nanoslit array for glancing angle transmission of light; the direct transmission through the dielectric layers is found to be strong, generating many side lobes of high intensity.

## Conclusions

We have developed a slanted-nanoslit array lens structure and demonstrate a subdiffraction-limited (*λ*/3) focusing of UV–visible light (325 nm or 633 nm) with focal length of 1–4*λ*. This sub-Abbe-limited focusing was enabled by manipulating the far-field transmission patterns of nanoslit apertures for more glancing-angle propagation, and thereby increasing the maximal in-plane wave-vectors. This method corresponds to altering the obliquity factor of wavelet propagation and offers a new degree of freedom in designing far-field super-resolution lenses.
